# MetaCRS: unsupervised clustering of contigs with the recursive strategy of reducing metagenomic dataset’s complexity

**DOI:** 10.1186/s12859-021-04227-z

**Published:** 2022-01-20

**Authors:** Zhongjun Jiang, Xiaobo Li, Lijun Guo

**Affiliations:** 1 College of Information Science and Technology, Ningbo University, Ningbo, 315211 China; 2 College of Mathematics and Computer Science, Zhejiang Normal University, Jinhua, 321004 China; 3 College of Engineering, Lishui University, Lishui, 323000 China

**Keywords:** Metagenomics, Unsupervised clustering, Contigs, Recursive strategy, Complexity of metagenomic samples

## Abstract

**Background:**

Metagenomics technology can directly extract microbial genetic material from the environmental samples to obtain their sequencing reads, which can be further assembled into contigs through assembly tools. Clustering methods of contigs are subsequently applied to recover complete genomes from environmental samples. The main problems with current clustering methods are that they cannot recover more high-quality genes from complex environments. Firstly, there are multiple strains under the same species, resulting in assembly of chimeras. Secondly, different strains under the same species are difficult to be classified. Thirdly, it is difficult to determine the number of strains during the clustering process.

**Results:**

In view of the shortcomings of current clustering methods, we propose an unsupervised clustering method which can improve the ability to recover genes from complex environments and a new method for selecting the number of sample’s strains in clustering process. The sequence composition characteristics (tetranucleotide frequency) and co-abundance are combined to train the probability model for clustering. A new recursive method that can continuously reduce the complexity of the samples is proposed to improve the ability to recover genes from complex environments. The new clustering method was tested on both simulated and real metagenomic datasets, and compared with five state-of-the-art methods including CONCOCT, Maxbin2.0, MetaBAT, MyCC and COCACOLA. In terms of the number and quality of recovered genes from metagenomic datasets, the results show that our proposed method is more effective.

**Conclusions:**

A new contigs clustering method is proposed, which can recover more high-quality genes from complex environmental samples.

## Background

Before the emergence of metagenomics technology, the related research on microorganisms was mainly through artificial pure culture of a single microorganism. However, most microorganisms are difficult or impossible to be cultured purely on the medium in the natural environment [1]. Metagenomics arises with the development of second-generation sequencing technology, which can obtain the genetic material of all microorganisms in the samples directly from the natural environments without the need for pure culture on the medium like the traditional methods. Metagenomics provides new research ideas for scientists to study the community structure of microbes, the interaction between microbes and the relationship between microbes and the environment or diseases [2]. The shotgun sequences obtained by second-generation sequencing can be assembled into longer gene fragment (contigs) by short reads assemblers [3, 4]. Due to the limitations of assembly tools, only scattered gene fragments, not complete genes can be assembled. The binning methods of contigs are subsequently used to obtain more complete genes from metagenomic datasets.

The existing metagenomic binning methods are generally divided into two types, supervised classification and unsupervised clustering methods [5]. The reads obtained by second-generation sequencing are very short, which are only 50 bp to 200 bp. They carry limited information, so it is difficult to classify them effectively [6–8]. As the accuracy of assembly tools increases, which can reach 97% [9–11], more and more methods are used to classify assembled contigs.

Supervised classification methods use known genes as references and classify the contigs based on the homology of gene sequence and similarity of sequence composition [12, 13]. Due to the need to build the reference databases and indexes, it requires high computer memory and hard disk storage space. In addition, there are a large number of unknown species in the environment, which cannot be matched with the sequences in the reference databases. Therefore, there will be a large number of unclassified contigs. Furthermore, the method based on the similarity of sequence composition is slow in modeling when faced with complex metagenomic samples, and it is difficult to obtain training data and labels. On the contrary, unsupervised clustering methods can use the composition information of the sequences themselves [14, 15], and their abundance information in samples [16–18] or both [19–22] to perform clustering in order to obtain the complete genes of unknown strains and discover new strains.

The current mainstream clustering methods include CONCOCT [20], Maxbin2.0 [23], MetaBAT [24], MyCC [25], COCACOLA [26], DAS tool [27], etc. CONCOCT uses the composition information of the sequence (tetranucleotide frequency) and co-abundance to vectorize all sequences, and then uses the Principal Component Analysis method to reduce their dimensionality. Gaussian mixture model combined with the Expectation Maximization Algorithm is used to classify contigs. CONCOCT performs well in the simple metagenomic datasets, but it performs poorly in the complex metagenomic datasets. Mabin2.0 and MetaBAT both combine sequence composition features and co-abundance, and calculate the probability of each sequence to the cluster centers through a pre-trained probability model. Then the Expectation Maximization algorithm and modified K-medoid algorithm are used for clustering respectively. Maxbin2.0 performs well on medium-complexity metagenomic datasets, but the ability to recover high-quality genes on the high-complexity metagenomic datasets may decrease, and it couldn’t be applied to the ultra-high complexity metagenomic datasets. MetaBAT is an algorithm specially designed for complex metagenomic datasets, which performs well on ultra-high complexity metagenomic datasets. However, the disadvantage is that the algorithm needs too many parameters to be adjusted for different datasets, otherwise the expected results cannot be achieved. MyCC combines genomic signatures, marker genes and optional contig coverages within one of multiple samples. It performs well on low complexity datasets, but the performance may decrease greatly on medium and high complexity datasets. COCACOLA uses $L_{1}$ distance instead of Euclidean distance as similarity measure, and combines the advantages of hard clustering and soft clustering through sparse regularization. In addition, COCACOLA also combines customized knowledge to improve clustering accuracy. Like most clustering methods, it can’t achieve good performance in complex environmental datasets. DAS tool is not an independent tool, and it is an integrated tool, and its performance is mainly determined by the performance of the tools it contains.

The main problems of the current unsupervised clustering methods include: (1) The ability to recover genes in complex environment needs to be improved. (2) The number of strains is a key parameter of the unsupervised clustering method which greatly affects the performance of the algorithm, but the selection of the number of strains in the clustering process is very different from the actual situation. (3) It is difficult to distinguish the sequences from the same species but different strains in the samples. There are two reasons. One reason is that it is easy to produce chimeras due to the high sequence similarity from the same species but different strains when using assembly tools to assemble the reads [9]. The second reason is that there are lots of species in a complex environment, which makes it difficult to distinguish effectively.

To address these problems, we propose a new clustering method MetaCRS (MetaCRS: unsupervised clustering of Contigs with the Recursive Strategy of reducing metagenomic dataset’s complexity) that can continuously reduce the complexity of the samples through a recursive strategy to improve the ability to recover genes from complex environments and a new method to determine the number of strains in the samples. We first pre-train the probability model which can calculate the probabilities that any two contigs come from the same strain. The probability model and composition characteristics of the sequences (tetranucleotide frequency) and co-abundance are used to calculate the probabilities between each contig and all cluster center sequences. We also propose a new method to determine the number of strains in the samples. After combing the sequences analyzed by a marker gene identification method [13, 28–33] and the gene sequences predicted by FragGeneScan [34], they will be screened and filtered. Then the sequences that can be used as the initial cluster centers are finally obtained, and their number is the initial value of the number of strains in the samples. A recursive strategy is adopted to continuously reduce the sample’s complexity to improve the ability to recover genes from metagenomic datasets.

## Methods

**Fig. 1:**
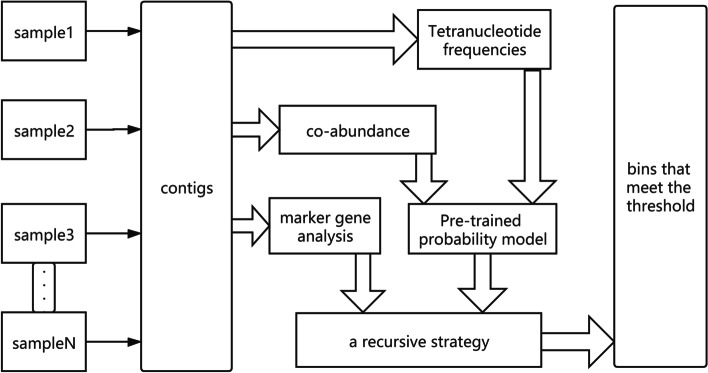
The whole pipeline of MetaCRS method

The whole pipeline of the method is shown in Fig. 1. Firstly, the reads of each sample are aggregated to build a gene library. Then they will be assembled into the contigs. The composition feature (tetranucleotide frequency) and co-abundance of the sequences are counted and combined with the pre-trained probability model to calculate the probabilities of which the sequences are from the same strain. Through the marker gene analysis, the parameters can be initialized in the clustering process, such as the number of clusters and the initial cluster center sequences. In the clustering process, we propose a new clustering method that can continuously reduce the complexity of the samples through a recursive strategy. Finally, the bins that meet the threshold can be obtained.

### Measuring probabilities of each contig belonging to any clustering center

We define a probability model which can calculate the probabilities of each contig belonging to any cluster centers based on their tetranucleotide frequencies and co-abundance [23, 24]. Tetranucleotide frequency is defined as the frequency of four consecutive nucleotides in a given gene sequence, and it is proved to have species-specific patterns in gene feature representation [35–38]. Meanwhile, co-abundance feature is proved to be very effective to deconvolute complex communities if there are many samples available [20, 21]. The 3181 bacterial and archaeal genes downloaded from the IMG website [22] are simulated to generate metagenomic datasets. The Euclidean distance is calculated between tetranucleotide frequencies extracted from intra-genome (sequences from the same gene) and inter-genome (sequence from different genes) sequences 1 million times in order to obtain the prior probability distribution of the Euclidean distance between tetranucleotide frequencies from the same gene and from different genes [22]. Then the posterior probability of two contigs from the same gene is calculated according to the following Bayesian formula [24]:

1 $$P_{{{\text{dist}}}} (R|D) = \frac{{P\left( R \right)P(D|R)}}{{P\left( R \right)P(D|R) + P\left( T \right)P(D|T)}}$$

where T represents the situation where two contigs are from different strains and R represents the situation where two contigs are from the same strains. D is the Euclidean distance between tetranucleotide frequencies of two contigs. Here we set $P\left( T \right) = 10*P\left( R \right)$.

The posterior probability between contigs of different lengths can be approximated by logistic regression as shown in (2):

2 $$P\left( {D_{{ij}} ;b_{{ij}} ,c_{{ij}} } \right) = \frac{1}{{1 + e^{{ - \left( {b_{{ij}} + c_{{ij}} *D_{{ij}} } \right)}} }}$$

where $D_{{{\text{ij}}}}$ represents the Euclidean distance between tetranucleotide frequencies of contig i and contig j. The b and c are two logistic regression parameters, which are estimated from experimental data [24].

Shotgun sequencing follows the Lander–Waterman model, which uses the Poisson distribution to calculate the coverage of contigs [39]. The Poisson distribution is used to evaluate the similarity between the sequence S and the cluster center G in the metagenomic sample k [22, 24]. The probability is defined as follows:

3 $$P_{{COV}} \left( {S \in G{\text{|}}cov\left( {G_{k} } \right)} \right) = Possion(cov\left( {S_{k} } \right)|cov\left( {G_{k} } \right)$$

where $\text{cov} (S_{k} )$ and $cov\left( {G_{k} } \right)$ is the coverage of sequence S and cluster center sequence G in metagenomic sample k, $~~P_{{COV}} \left( {S \in G{\text{|}}cov\left( {G_{k} } \right)} \right)$ is a Poisson probability density function given mean $\lambda = {\text{cov}}\left( {G_{k} } \right)$.

Assuming that all metagenomic samples are independently sequenced, the similarity probability of coverage between sequence S and cluster center sequence G needs to consider all metagenomic samples, which is defined as:

4 $$\mathop \prod \limits_{{k = 1}}^{M} P_{{cov}} \left( {S \in G{\text{|}}cov\left( {G_{k} } \right)} \right) = ~\mathop \prod \limits_{{k = 1}}^{M} Possion(cov\left( {S_{k} } \right)|cov\left( {G_{k} } \right))$$

where M is the number of metagenomic samples, $cov\left( {S_{k} } \right)$ and $cov\left( {G_{k} } \right)$ is the coverage of sequences S and G in metagenomic sample k.

$P_{{{\text{dist}}}} ~~$ and $P_{{cov}}$ are combined as a measure of the probabilities between each config and cluster center sequences, which is defined as:

5 $$P\left( {S \in G} \right) = P_{{dist}} \left( {R{\text{|}}D} \right) \cdot \mathop \prod \limits_{{k = 1}}^{M} P_{{cov}} \left( {S \in G{\text{|}}cov\left( {G_{k} } \right)} \right)$$

Formula (5) is the probability model used in the following clustering process.

### A recursive strategy for clustering

**Fig. 2:**
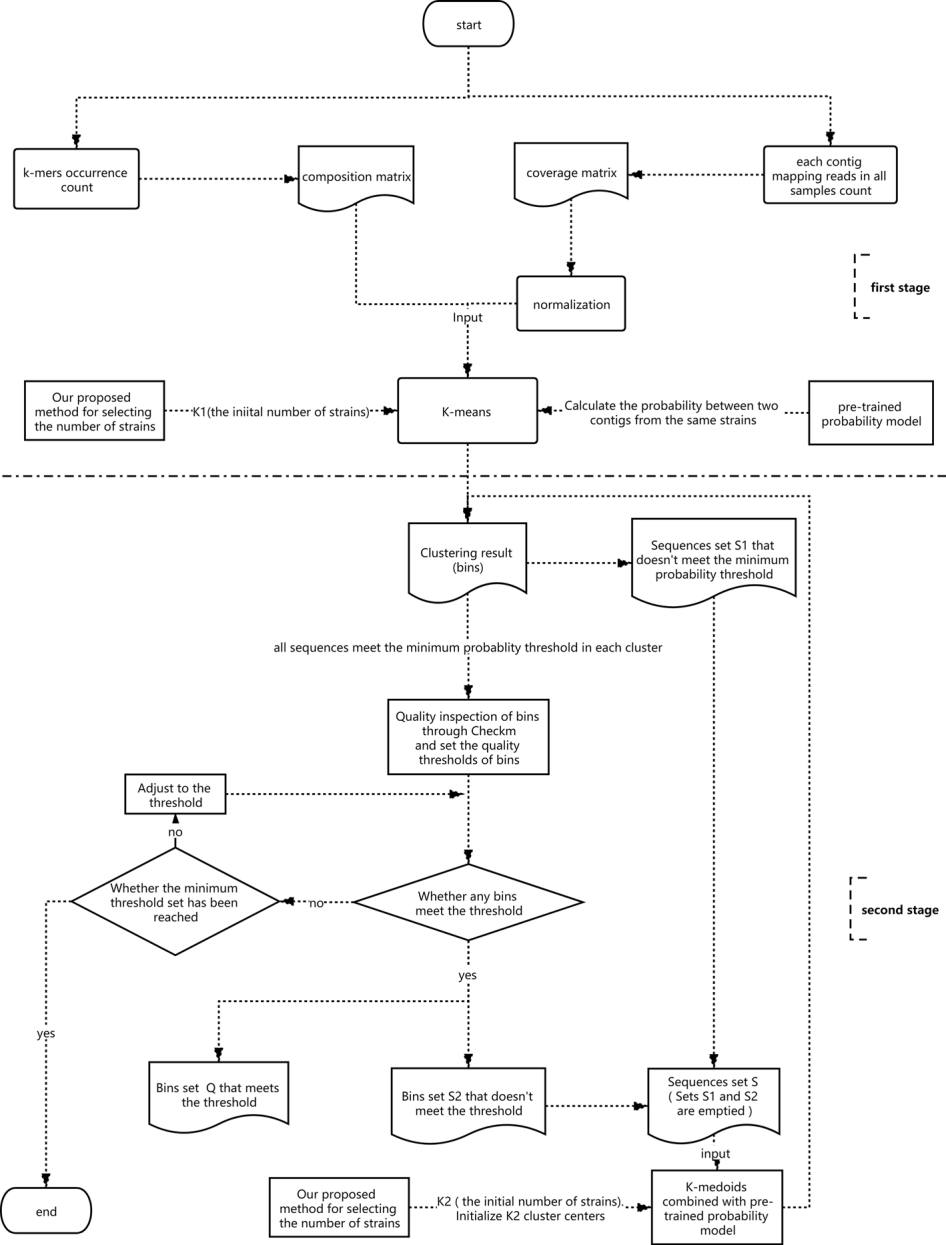
The whole pipeline of recursive strategy for clustering

A recursive strategy is proposed for clustering, which can continuously reduce the complexity of samples to improve the ability to recover genes from complex environmental samples. The whole pipeline of the algorithm is shown in Fig. 2. It is mainly divided into the following two stages. At the first stage, for each contig in the dataset, their tetranucleotide frequencies and co-abundance are calculated first to form the composition matrix and coverage matrix. The coverage matrix is normalized due to the different lengths of contigs, so as that our pre-trained probability model can calculate probabilities of the contigs in different lengths. Because the K-means algorithm [40] can converge quickly and dirty data has little effect under large datasets, we use the K-means algorithm combined with the pre-trained probability model to cluster.

At the second stage, the clustering results are processed first. We filter out the sequences that don’t meet the minimum probability threshold in each cluster and put them in the set S1. Then we use the quality evaluation tool CheckM [41] to detect the quality of the bins and set the quality thresholds. If there are no bins that meet the threshold, it is determined whether the minimum threshold setting is reached. If the minimum threshold setting has been reached, the algorithm ends. Otherwise the threshold is adjusted and continues to determine whether there are bins that meet the threshold. If there are bins that meet the threshold, put them into the set Q and put the bins that don’t meet the threshold into the set S2. Then contigs in sets S1 and S2 are mixed into set S, and S1 and S2 are cleared at the same time. K-medoids algorithm [42] is used to cluster the set S. The initial number K2 of strains is given and K2 cluster centers are initialized by our proposed method for selecting the number of strains in the set S. Then the second stage is repeated. Among them, CheckM is used to calculate the recall (percent of expected single-copy-genes that are binned) and precision (the absence of genes from different genomes) rates. The precision is estimated from the number of multicopy marker genes identified in each marker set [41]:

6 $$precision = \frac{{\mathop \sum \nolimits_{{s \in M}} \frac{{\mathop \sum \nolimits_{{g \in s}} C_{g} }}{{\left| s \right|}}}}{{\left| M \right|}}$$

where s is a set of collocated marker gene and M is the set of all collocated marker sets s. $C_{g}$ is N−1 for a gene g identified $N \ge 1$ times, and 0 for a missing gene.

The recall is estimated as the number of marker sets present in a genome taking into account that only a portion of a marker set may be identified [41]:

7 $${\text{recall}} = \frac{{\mathop \sum \nolimits_{{s \in M}} \frac{{\left| {s \cap G_{M} } \right|}}{{\left| s \right|}}}}{{\left| M \right|}}$$

where s is a set of collocated marker gene. M is the set of all collocated marker sets s, and $G_{M}$ is the set of marker genes identified in a genome*.*

The overall procedure is summarized as follows.

*The first stage of the algorithm:*

1. *Compute the composition and coverage matrices.*
2. *Normalize the coverage matrix.*
3. *Estimate the number of clusters K1. through our proposed method.*
4. *Clustering contigs by K-means:*
  - *Initialization: randomly select K1 contigs as the cluster centers.*
  - *Assignment step: associate each contig to the cluster center with the highest probability through the pre-trained probability model.*
  - *Update step: update the cluster centers by using the centroid of each cluster.*
  - *Repeat steps b and c until there is no change of the cluster centers.*
*The second stage of the algorithm:*
5. *Filter out the sequences that don’t meet the minimum probability threshold in each cluster and put them in the set S1.*
6. *Detect the quality of each bin and set the quality thresholds (set thresholds when you meet for the first time, skip it when you meet again) through CheckM.*
7. *Determine whether there are bins that meet the threshold. If not, it is judged whether the minimum threshold setting has been reached. If it is reached, the algorithm ends, otherwise the threshold setting is adjusted and the judgment is made again. If there are bins that meet the threshold, go to step 8.*
8. *Put the bins that meet the threshold into the set Q and put the bins that don’t meet the threshold into the set S2. Mix the set S1 and S2 into set S. Clear S1 and S2 at the same time.*
9. *Clustering contigs in set S by K-medoids:*
  1. *Initialization* *:* *The initial number K2 of strains is given and K2 medoids are initialized through our proposed method for selecting the number of strains in the set S.*
  2. *Assignment step: associate each contig to the medoid with the highest probability through the pre-trained probability model.*
  3. *Update step* *:* *For all other contigs in each cluster except the corresponding medoid, the value of the criterion function is calculated when they become a new medoid in order. The sequence is selected as a new medoid corresponding to the minimum value of the criterion function. The criterion function based on the average dissimilarity of all contigs to the new medoid in the cluster.*
  4. *Repeat steps (2) and (3) until there is no change of the medoids.*
10. *Back to step 5.*

### Estimation of the number of strains and Initialization of the algorithm

**Fig. 3:**
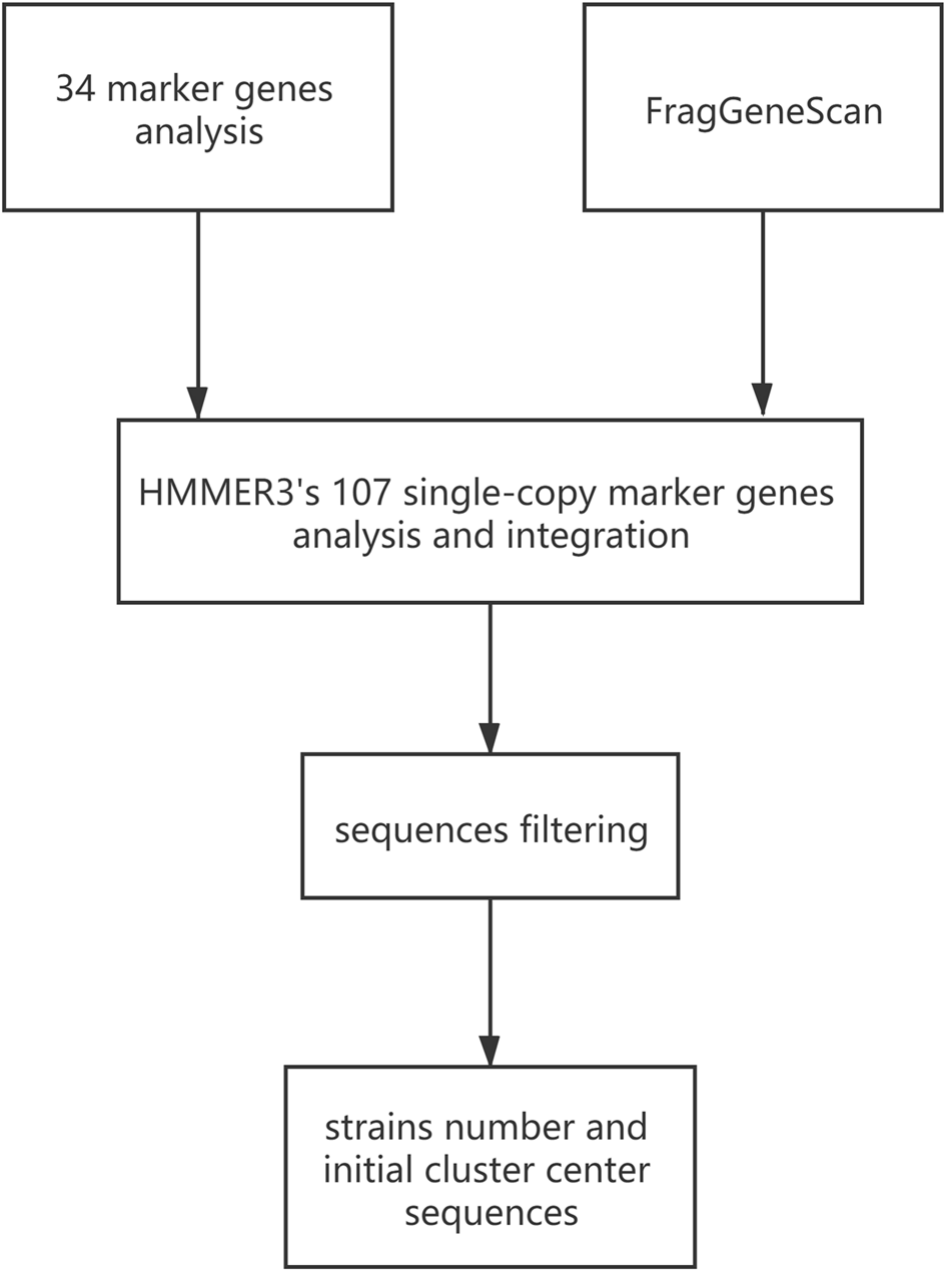
The method of selecting the number of strains in the sample and the method of clustering initialization

We propose a new method for selecting the number of strains in metagenomic datasets. Six reading frames are used to translate the DNA sequences in the dataset into the protein sequences. Among all the sequences that have been translated or not, the DNA sequences and Hidden Markov model profiles of 34 marker genes with classification information are identified [13, 28–33]. The recognized DNA sequences combined with the genes predicted by FragGeneScan [34] are used to make a de-redundant integration. Then HMMER3 [43] are applied to analyze the integrated genes with 107 single-copy marker genes, and they are filtered to obtain the shortest number of marker gene sequences, which is defined as the number of strains, and these sequences are used as the initial cluster centers [22]. As shown in Fig. 3, the K1 and K2 parameters of the clustering algorithm are obtained, and the cluster centers of the second stage in clustering algorithm are initialized with the sequences obtained by this method.

### Definition of the complexity of metagenomic samples

The concept of complexity is defined as follows when the metagenomic datasets are constructed [9, 23, 24]:

8 $$Metacomplexity = GN + CN$$

where $Metacomplexity$ represents the complexity of the metagenomic datasets. $GN$ represents the number of genes and $CN$ represents the circular elements that are distinct from strains, species, genera or orders represented by public genomes in the metagenomic datasets. Here in the Metacomplexity value between 0 and 200 is considered as low complexity, between 200 and 800 as medium complexity, between 800 and 1400 as high complexity, and above 1400 is considered as ultra-high complexity.

## Results

In order to verify the effectiveness of our proposed method and the ability to recover genes from complex environments, we compared it with five state-of-the-art methods including CONCOCT, Maxbin2.0, MetaBAT, MyCC, and COCACOLA on simulated and real datasets.

### Binning performance on CAMI simulated datasets

The simulation datasets in [9] was used for benchmark testing, which was generated to have a unified evaluation standard for each clustering algorithm. The simulation datasets were divided into low complexity dataset (40 genomes and 20 circular elements), medium complexity dataset (132 genomes and 100 circular elements), and high complexity dataset (596 genomes and 478 circular elements). These datasets were from newly sequenced genome of about 700 microbial isolates and 600 circular elements that were distinct from strains, species, genera or orders represented by public genomes. At the same time, they are consistent with the situation in the real environment, including a large number of closely related strains, plasmids, viral sequences, and realistic abundance profiles.

**Fig. 4:**
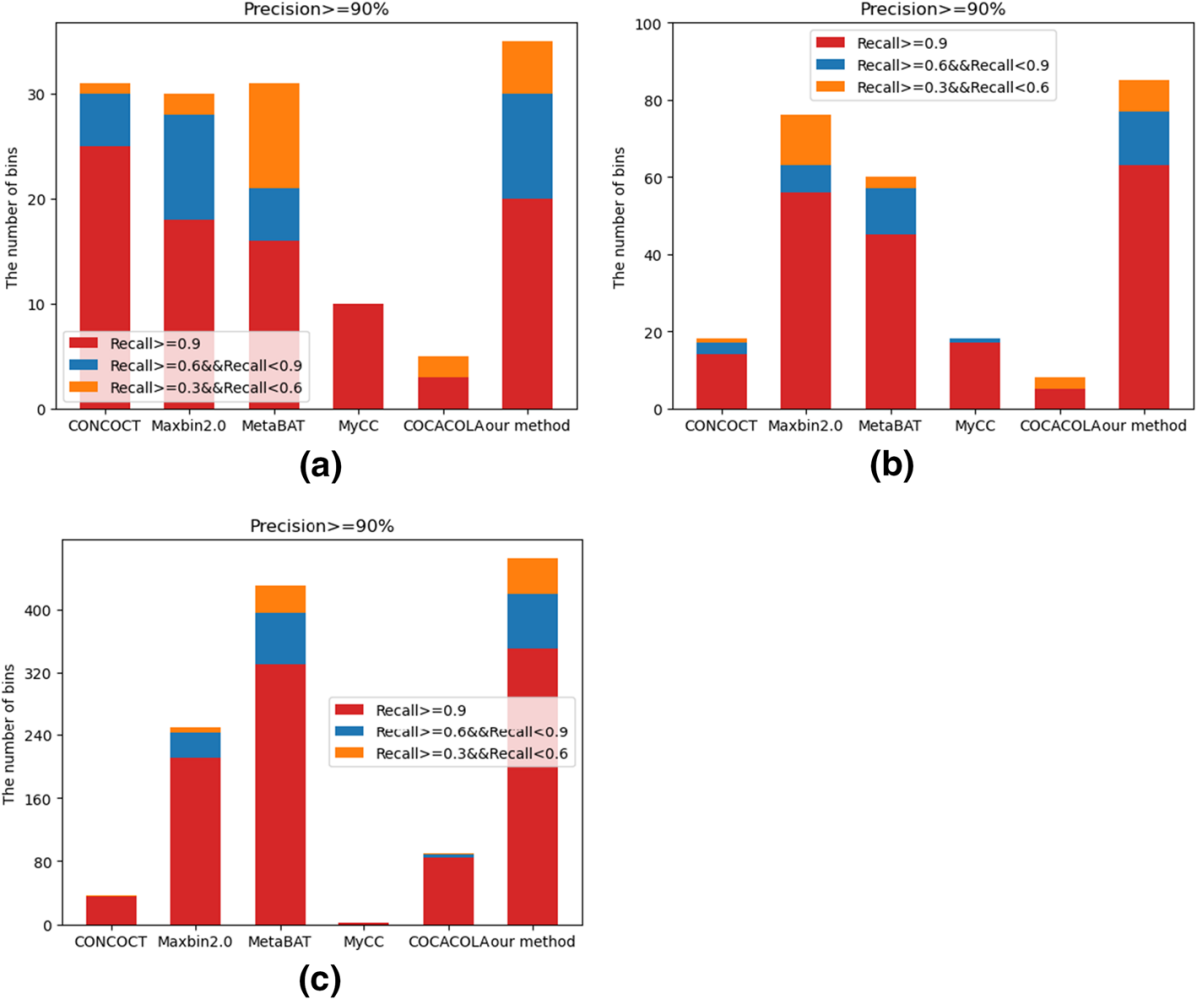
The performance of CONCOCT, Mabin2.0, MetaBAT, MyCC, COCACOLA and our proposed method on the CAMI’s datasets with low-complexity (**a**), medium-complexity (**b**) and high-complexity (**c**)

We tested our method on three datasets of different complexity, and compared it with other state-of-the-art clustering methods including CONCOCT [20], Maxbin2.0 [23], MetaBAT [24], MyCC [25], and COCACOLA [26]. The number of bins was counted with the precision rate greater than 90% and the recall rate greater than 30% (bins that meet this condition are considered as good quality bins, and it is generally believed that the bins are different strains from the same species.). Since the composition characteristics on short contigs were not obvious, it would affect the clustering effect. Here we clustered contigs larger than 1500 bp, and the contigs shorter than 1500 bp were excluded. The minimum probability threshold of all contigs to the cluster center in each cluster after clustering was set as 80%, and there were three threshold conditions for bins screening using CheckM [41]: the precision rates were set to be greater than 90%, and the recall rates were set to be greater than 90%, 60%, and 30% respectively. The number of bins obtained were counted. As show in Fig. 4. Our proposed method obtained the largest number of genes in almost every recall threshold both in medium-complexity and high-complexity dataset. Especially in high-complexity datasets, our method performed much better than the other five methods. In low-complexity dataset, CONCOCT was better than our method at the recall rate greater than 90%. This may be the reason that the K-means algorithm of our proposed method is affected by dirty data in low-complexity dataset, resulting in poor clustering effect in the first stage. As shown in Table 1, our method identified the largest number of recovered genes when the precision rate was greater than 90% and the recall rate was greater than 30%. When both the precision rate and the recall rate were greater than 95%, the number of recovered genes was only less than CONCOCT on the low-complexity dataset and was the largest on the other datasets.

**Table 1:** The number of strains recovered by the six clustering methods

Methods	Low complexity	Medium complexity	High complexity
CONCOCT	14/17	10/18	24/36
Maxbin2.0	11/22	42/76	155/250
MetaBAT	10/18	40/63	235/440
MyCC	10/10	16/18	2/2
COCACOLA	1/5	1/8	55/90
Our method	12/25	55/97	263/472

The left side of/is the number of bins recovered when precision rate is greater than 95% and recall rate is greater than 95%. The right side of/is the number of bins recovered when precision rate is greater than 90% and recall rate is greater than 30%

### Binning performance on real metagenomic assembly datasets

**Fig. 5:**
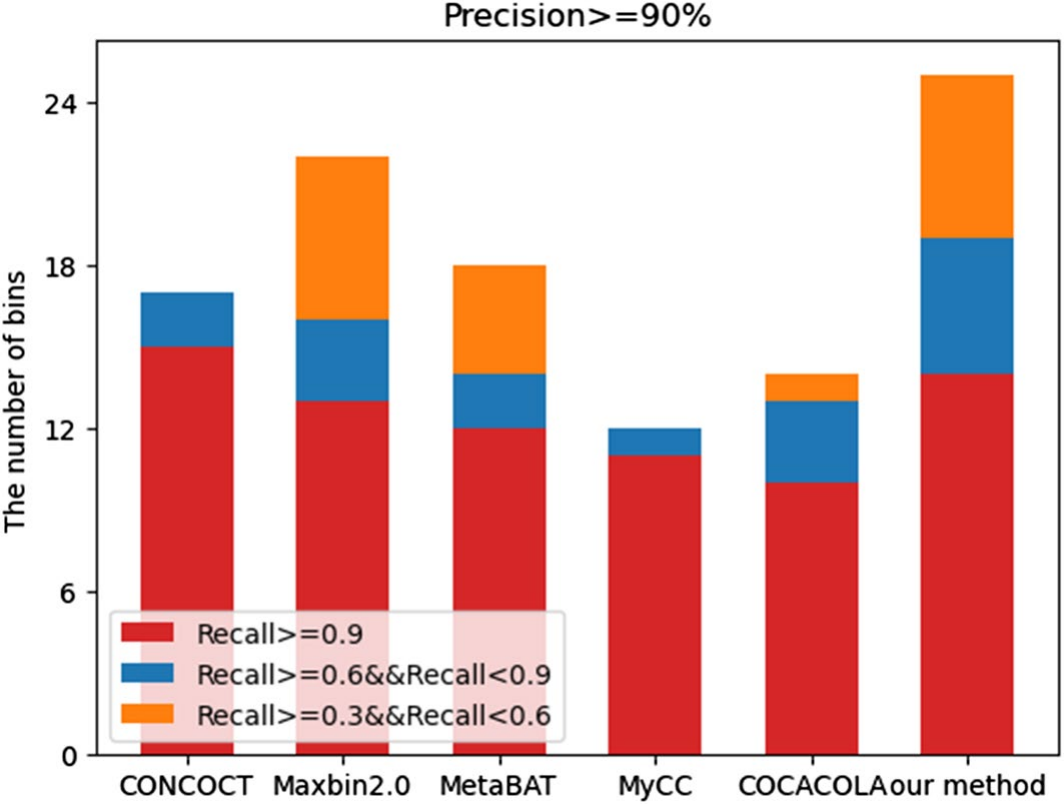
The performance of CONCOCT, Maxbin2.0, MetaBAT, MyCC, COCACOLA and our proposed method on the Sharon dataset. The x-axis represents six different clustering methods, and the y-axis represents the number of strains obtained with different recall rate thresholds when the precision rate was greater than 90%

We compared our proposed method with the other five methods on the Sharon dataset [44]. The Sharon dataset contains 2329 assembled contigs, which comes from gut microbes of an obese human. We filtered out the contigs that did not exceed 1500 bp, and mapped reads to contigs through Bowtie2 [31] to get the coverage information of each contig. We ran six methods on this dataset, and the parameter settings were the same as the simulation datasets. As shown in Fig. 5, our method performed better than the other five methods, and identified the highest number of bins between 90 and 60% of the recall rate. CONCOCT obtained the highest number of bins when the recall rate was greater than 90%.

**Fig. 6:**
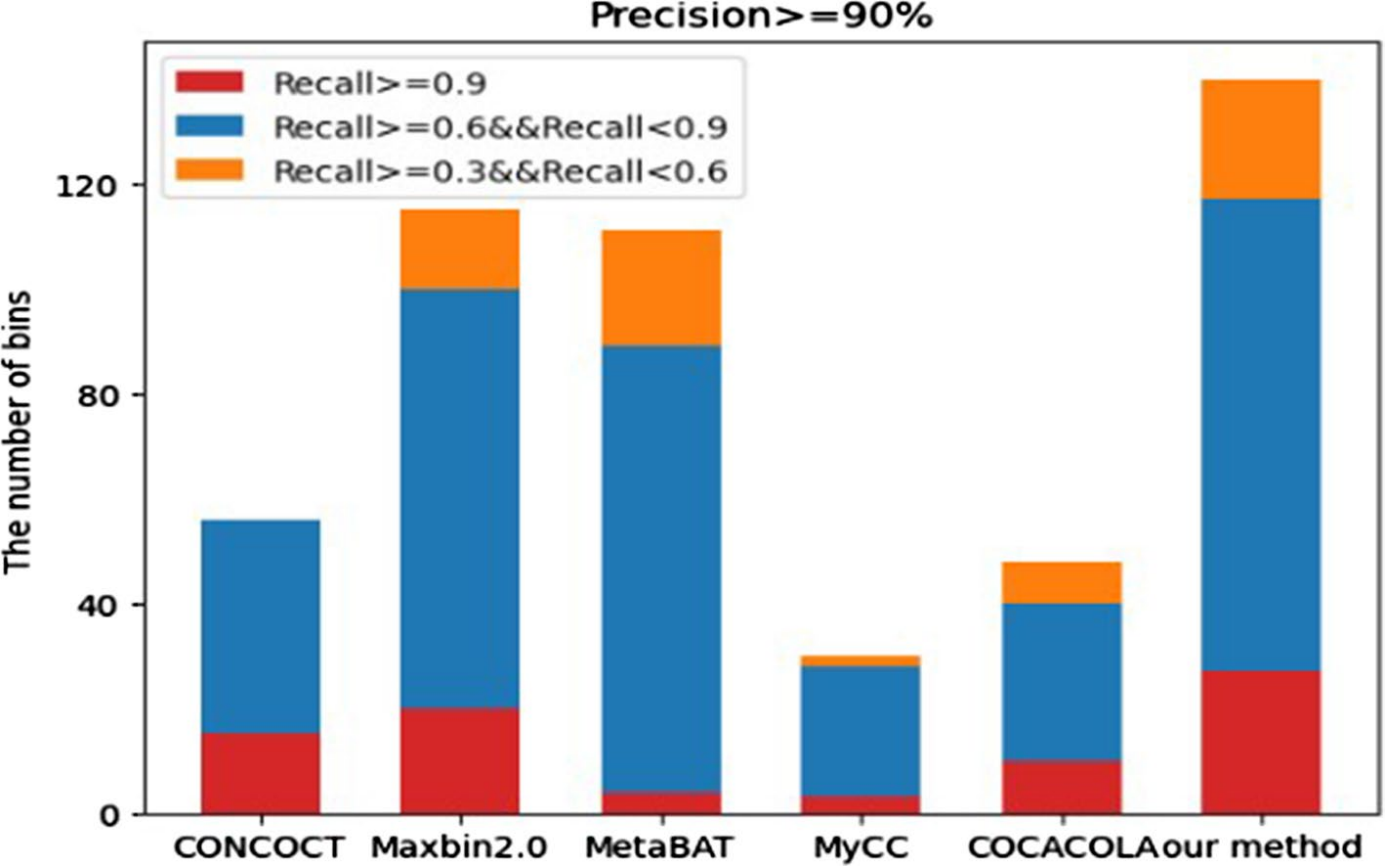
The performance of CONCOCT, Maxbin2.0, MetaBAT, MyCC, COCACOLA and our proposed method on the MetaHIT dataset. The x-axis represents six different clustering methods, and the y-axis represents the number of strains with different recall rate thresholds when the precision rate was greater than 90%

We tested all the six methods on the real dataset constructed in [24], which was from 264 MetaHIT human intestinal metagenomic data. Firstly, all data was selected from 264 MetaHIT human intestinal metagenomic data. After mapping all the reads to the bacterial genes in the NCBI library, 290 genes were selected with an average coverage greater than 5X and then the scaffolds of selected genomes were shredded using truncated exponential distribution of minimum contig size of 2.5 kb with 31 overlapped bases. Bowtie2 was used to match reads to each library. The coverage information of contigs was calculated, and 118,025 contigs was obtained for clustering. CheckM [41] was used to detect the quality of each bin. The results are shown in Fig. 6, our method achieved best results among other methods at each recall threshold similar to the simulated datasets situation, especially on the bins with the recall rate between 60 and 90%.

## Discussions

The pipeline of all of methods mainly consists of three modules, assembling short reads into contigs, binning contigs and evaluating clustering results. We mainly compared the running time of different algorithms for clustering. All of these algorithms were run on 8 virtual CPU and 32 GB-RAM cloud computing platform provided by Elastic Compute Service. The running time of binning between our method and five state-of-the-art methods including CONCOCT, Maxbin2.0, MetaBAT, MyCC and COCACOLA was compared on the simulated datasets and the real datasets. As shown in Table 2, MetaBAT and COCACOLA were faster than the other methods. Our proposed method was faster than Maxbin2.0. Although it took more time in the high complexity datasets, the running time was still acceptable compared to the high-quality genes identified. To reduce the time complexity, an alternative algorithm can be chosen [45] to replace the K-means algorithm.

**Table 2:** Running time of CONCOCT, Maxbin2.0, MetaBAT, MyCC, COCACOLA, MetaCRS (h:min:s)

Methods	‘Low’	‘Medium’	‘High’	‘Sharon’	‘MetaHIT’
CONCOCT	00:01:35	00:03:23	00:30:16	00:00:26	01:20:03
Maxbin2.0	00:10:03	00:16:24	02:45:03	00:01:45	06:35:36
MetaBAT	00:00:10	00:00:25	00:01:55	00:00:23	00:12:35
MyCC	00:00:13	00:00:59	00:10:36	00:00:16	00:15:26
COCACOLA	00:00:15	00:00:20	00:03:17	00:00:14	00:04:34
MetaCRS	00:05:24	00:11:45	02:13:52	00:00:56	05:26:43

## Conclusion

Reconstructing as many complete genes as possible from complex environments is still a hot topic in metagenomic research. In this paper, we propose a new clustering method which is based on feature vectorization of tetranucleotide frequency and co-abundance. A pre-trained probability model is used to implement the clustering process by using K-means [40] and a recursive strategy combining with K-medoids [42] algorithms and CheckM [41]. CheckM is a quality assessment tool for bins, and here it is used to simplify the complexity of the samples through a recursive strategy so that the clustering can achieve better results. We also propose a new method of selecting the number of strains in the samples. The key point of K-means and K-medoids algorithm lies in the selection of the number K of strains in the samples. Other methods such as HDBSCAN [45] don’t need to know the number of clusters in advance, but they need other parameters. We adopt the K-means algorithm in the first stage of the algorithm. Due to the high data dimensions, the time cost will increase when facing large-scale datasets. There are other alternative methods to replace K-means to reduce the time cost of the algorithm, such as stratified angle regression algorithm proposed in [46].

We tested our proposed method on simulated datasets and real datasets, and compared it with five state-of-the-art clustering methods including CONCOCT, MetaBAT, Maxbin2.0, MyCC and COCACOLA. Our proposed method achieved better performance in terms of precision, recall and estimated number of strains on both simulated and real datasets, and identified more high-quality genes in complex environmental samples. The main contributions of our work are: (1) A new recursive strategy is proposed, which could continuously reduce samples complexity and improved clustering performance. (2) A new method of selecting the number of strains in the samples is proposed. (3) Samples in natural environments are usually very complex, and our method performs much better in complex environments.

The use of assembly tools will bring chimeras of different strains from the same species. With the development of third-generation sequencing technology, it is expected that longer read fragments can be obtained quickly and cheaply. Clustering of longer reads will recover more high-quality genes from the complex environments. Next, we will apply this algorithm to the datasets containing long reads, and the effect may be better. At the same time, we will also study the clustering algorithm based on density [45] and consider to replace K-means with different clustering methods.

## Data Availability

The CAMI simulation datasets are available for academic use at https://data.cami-challenge.org/participate. The Sharon real dataset is available at http://ggkbase.berkeley.edu/carrol/. The MetaHIT real dataset is available at https://portal.nersc.gov/dna/RD/Metagenome_RD/MetaBAT/Files/MetaHIT/.

## Abbreviations

CONCOCT: Binning metagenomic contigs by coverage and composition
MaxBin 2.0: An automated binning algorithm to recover genomes from multiple metagenomic datasets
MetaBAT: An efficient tool for accurately reconstructing single genomes from complex microbial communities
MyCC: Accurate binning of metagenomic contigs via automated clustering sequences using information of genomic signatures and marker genes
COCACOLA: Binning metagenomic contigs using sequence COmposition, read CoverAge, CO-alignment and paired-end read LinkAge
MetaCRS: Unsupervised clustering of contigs with the recursive strategy of reducing metagenomic dataset’s complexity
